# The Evolving Understanding and Approach to Residual Cardiovascular Risk Management

**DOI:** 10.3389/fcvm.2020.00088

**Published:** 2020-05-13

**Authors:** Devinder S. Dhindsa, Pratik B. Sandesara, Michael D. Shapiro, Nathan D. Wong

**Affiliations:** ^1^Division of Cardiology, Department of Medicine, Emory Clinical Cardiovascular Research Institute, Atlanta, GA, United States; ^2^Section on Cardiovascular Medicine, Center for the Prevention of Cardiovascular Disease, Wake Forest Baptist Medical Center, Winston-Salem, NC, United States; ^3^Heart Disease Prevention Program, Division of Cardiology, University of California, Irvine, Irvine, CA, United States

**Keywords:** residual risk, secondary prevention, primary prevention, inflammation, cardiovascular risk factors, cardiovascular disease, atherosclerosis, metabolic syndrome

## Abstract

Despite unprecedented advances in treatment of atherosclerotic cardiovascular disease, it remains the leading cause of death and disability worldwide. Treatment of major traditional risk factors, including low-density lipoprotein-cholesterol, serves as the foundation of atherosclerotic risk reduction. However, there remains a significant residual risk of cardiovascular events despite optimal risk factor management. Beyond traditional risk factors, other drivers of residual risk have come to the forefront, including inflammatory, pro-thrombotic, and metabolic pathways that contribute to recurrent events and are often unrecognized and not addressed in clinical practice. This review will explore the evidence linking these pathways to atherosclerotic cardiovascular disease and potential future therapeutic options to attenuate residual cardiovascular risk conferred by these pathways.

## Introduction

The treatment of cardiovascular disease has seen unprecedented progress in the past several decades. Critical advancements have been made in the recognition and treatment of major traditional risk factors for atherosclerotic cardiovascular disease (ASCVD), among them elevated low-density lipoprotein (LDL)-cholesterol (LDL-C) levels, one of the principal drivers of atherosclerosis. An LDL-centric approach to risk reduction, namely with statins, has served as the foundation for primary and secondary prevention for decades and has led to significant improvement in cardiovascular outcomes. The development of the proprotein convertase subtilisin/kexin type 9 (PCSK9) inhibitors (PCSK9i) has driven LDL-C to levels previously not achievable by statins alone and the randomized controlled trials with PCSK9i provided further confirmation of the LDL hypothesis. Moreover, the cardiovascular outcomes trials with PCSK9i demonstrated that there was no level of achieved LDL-C that was not associated with further benefit. However, despite reduction of LDL-C levels to a mean of 30 mg/dL in the FOURIER (Further Cardiovascular Outcomes Research with PCSK9 Inhibition in Subjects with Elevated Risk) trial, the absolute risk reduction was only modestly reduced by 1.5%. In fact, the recurrent cardiovascular event rate at 3 years in those treated with a PCSK9i remained high at 9.8% (1). A similar finding was seen in ODYSSEY OUTCOMES (Evaluation of Cardiovascular Outcomes After an Acute Coronary Syndrome During Treatment with Alirocumab), in which the composite primary end point of cardiovascular death, non-fatal myocardial infarction (MI), stroke or unstable angina occurred in 9.5% of participants receiving alirocumab, as compared to 11.1% in the placebo arm, despite reductions of LDL-C levels to a mean of 40 mg/dL in the treatment group (2).

**Figure 1:**
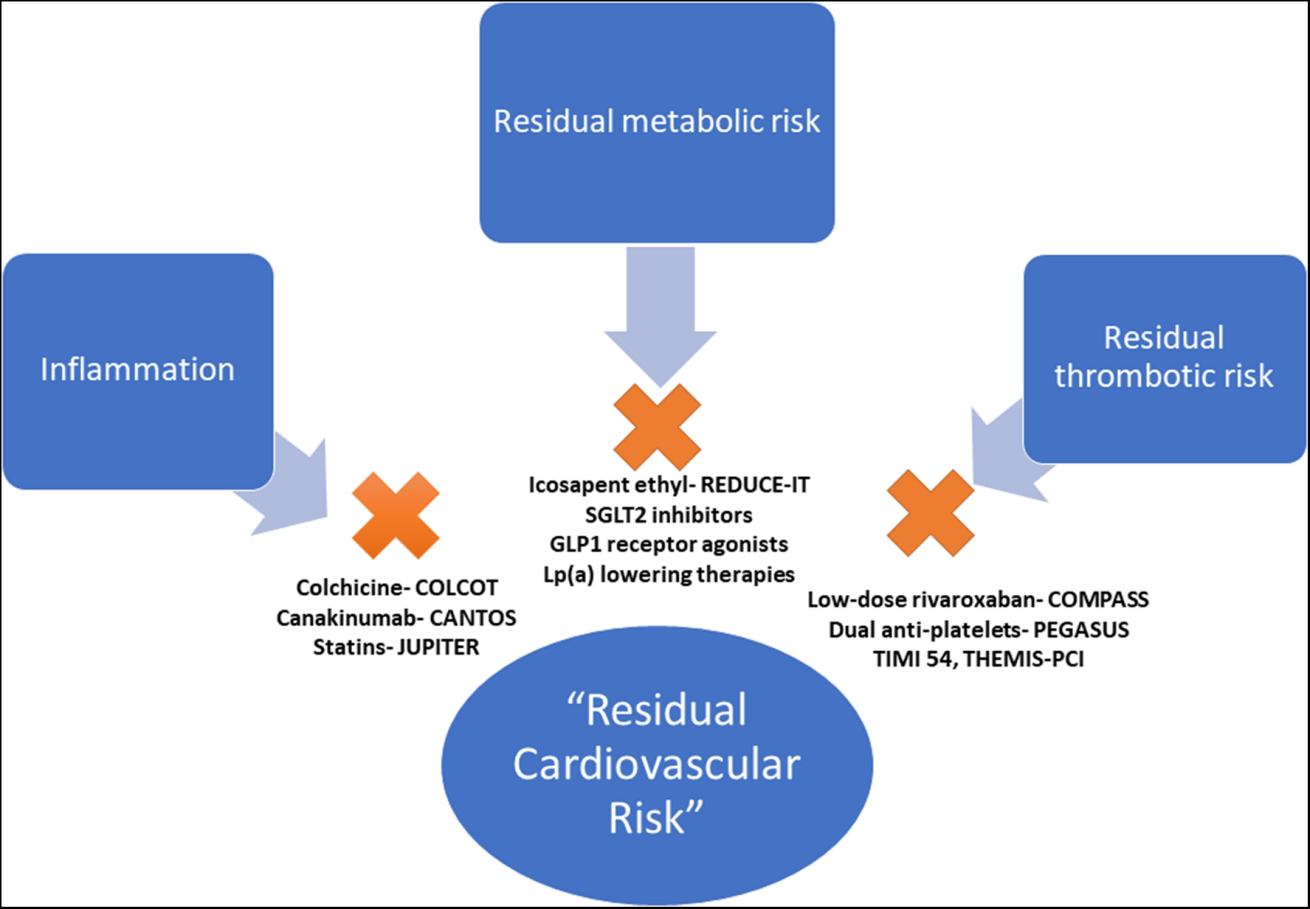
Pathways of residual cardiovascular risk. The pathways of residual cardiovascular risk, beyond traditional risk factors, with evidence-based therapeutic options. COLCOT, Colchicine Cardiovascular Outcomes Trial; CANTOS, Canakinumab Anti-inflammatory Thrombosis Outcomes Study; JUPITER, Justification for the Use of Statins in Prevention: an Intervention Trial Evaluating Rosuvastatin; PEGASUS TIMI 54, Prevention of Cardiovascular Events in Patients with Prior Heart Attack Using Ticagrelor Compared to Placebo on a Background of Aspirin–Thrombolysis In Myocardial Infarction 54; THEMIS-PCI, The Effect of Ticagrelor on Health Outcomes in Diabetes Mellitus Patients Intervention Study- PCI; COMPASS, Cardiovascular Outcomes for People Using Anticoagulation Strategies; REDUCE-IT, Reduction of Cardiovascular Events With EPA–Intervention; EMPA-REG, Empagliflozin, Cardiovascular Outcomes, and Mortality in Type 2 Diabetes trial; CREDENCE, Canagliflozin and Renal Events in Diabetes and Nephropathy Clinical Evaluation; DECLARE TIMI 58, Dapagliflozin Effect on Cardiovascular Events A Multicenter, Randomized, Double-Blind, Placebo-Controlled Trial to Evaluate the Effect of Dapagliflozin 10 mg Once Daily on the Incidence of Cardiovascular Death, Myocardial Infarction or Ischemic Stroke in Patients With Type 2 Diabetes; LEADER, Liraglutide Effect and Action in Diabetes: Evaluation of Cardiovascular Outcome Results.

The high observed event rate despite aggressive secondary prevention efforts speaks to the concept known as “residual cardiovascular risk,” defined as any suboptimally controlled causal risk factor for ASCVD. There are myriad drivers of residual risk, including pathways that are not directly addressed through current management strategies. Although treatment of LDL-C is the cornerstone of risk reduction, there are other drivers of ASCVD and recurrent events. Although residual risk can be attributable to many factors (i.e., hypertension, tobacco use, elevated blood glucose), this review will focus on pathways beyond traditional risk factors addressed in usual clinical care (inflammatory, pro-thrombotic, and metabolic) that may play a critical role in driving recurrent cardiovascular events (Figure 1).

## Residual Inflammatory Risk

Beyond LDL-C lowering, statins are known to have multiple pleiotropic effects. Of note, numerous trials demonstrated consistent anti-inflammatory effects. In the JUPITER trial (Justification for the Use of Statin in Prevention: an Interventional Trial Evaluating Rosuvastatin), rosuvastatin 20 mg daily reduced median high sensitivity C-reactive protein (hsCRP) by 37% as compared to placebo (3). Interestingly, the magnitude of hsCRP reduction achieved with rosuvastatin was proportional to the reduction in cardiovascular risk. Individuals who achieved hsCRP levels <2 mg/L demonstrated a 55% reduction in the primary endpoint compared to those with hsCRP levels ≥2 mg/L (*p* = 0.007); however, this benefit was not independent of LDL-C lowering. Similarly, in PROVE-IT TIMI 22 (Pravastatin or Atorvastatin Evaluation and Infection Therapy-Thrombolysis in Myocardial Infarction 22), those who achieved hsCRP levels <2 mg/L sustained fewer recurrent cardiovascular events (4). Whether the benefits of statins are related to LDL-C lowering, reduction in inflammation, or a combination of these factors remains a matter of debate.

In addition to hsCRP, one biomarker of inflammation, lipoprotein-associated phospholipase A2 (Lp-PLA2), is known to increase the production of pro-inflammatory and proapoptotic mediators within atherosclerotic plaques (5–10). In observational studies, the increase in Lp-PLA2 was associated with increased risk of adverse cardiovascular outcomes (6, 11). However, when a potent inhibitor of Lp-PLA2, darapladib, was tested in a randomized controlled trial in a cohort with stable coronary heart disease, there was no benefit seen in cardiovascular outcomes (12). Importantly, 96% of patients enrolled in the trial were on statins, which themselves are known to reduce LP-PLA2 by 35% (13–15). This inhibition of Lp-PLA2 with associated reduction of inflammation and plaque stabilization may be one of the several mechanisms through which statins exert their benefit.

The direct causal role of inflammation in cardiovascular disease was not formally proven until CANTOS (Canakinumab Anti-Inflammatory Thrombosis Outcome Study) (16). CANTOS enrolled 10,061 participants with a history of myocardial infarction (MI), optimized LDL-C, and hsCRP ≥ 2 mg/L and randomized them to optimal medical therapy (OMT) plus placebo vs. OMT plus canakinumab, a fully human monoclonal antibody targeted to interleukin-1β (IL-1β). Importantly, previous studies demonstrated that canakinumab has no effect on LDL-C. For the primary efficacy end-point of non-fatal myocardial infarction (MI), non-fatal stroke, or cardiovascular death, there was benefit observed with the 150 mg dose (HR 0.85, 95% CI 0.74–0.98, *p* = 0.021) and 300 mg dose (0.86 95% CI 0.75–0.99, *p* = 0.031) of canakinumab as compared to placebo. Importantly, lowering of hsCRP to levels <2 mg/L lead to a 25% reduction in major adverse cardiovascular events (MACE) and a 31% reduction in cardiovascular death and all-cause mortality, without any effect on LDL-C. There were non-significant reductions in mortality if hsCRP levels remained above 2 mg/L. A surprising finding was the reduction in cancer mortality associated with high dose (300 mg) canakinumab compared to placebo (HR 0.49, 95% CI 0.31–0.75; *p* = 0.0009), lung cancer mortality (HR 0.23, 95% CI 0.10–0.54; *p* = 0.0002), and incident lung cancer (HR 0.33, 95% CI 0.18–0.59; *p* < 0.0001), lending credence to the hypothesis that inflammation also plays a central role in the evolution of malignancy (17). Regarding safety, neutropenia and death due to sepsis were more common in the treatment arm than placebo (incidence rate 0.31 vs. 0.18 events per 100 person-years; *p* = 0.02). The FDA did not grant canakinumab an indication for cardiovascular risk reduction.

The Cardiovascular Inflammation Trial (CIRT) also sought to investigate the role of inflammation reduction in mitigating cardiovascular disease risk. In CIRT, over 3,000 subjects with a history of MI or multivessel coronary artery disease as well as type 2 diabetes mellitus or metabolic syndrome were randomized to OMT plus placebo vs. OMT plus low dose methotrexate (15–20 mg weekly) (18). In the treatment arm, there was no effect on cardiovascular events or all-cause mortality with low-dose methotrexate as compared to placebo. Importantly the median hsCRP in this trial was 1.5 mg/L at baseline, and at 8 months following randomization, there was no impact on blood levels of hsCRP, interleukin (IL)-6, or IL-1β. Taking the findings of CANTOS and CIRT together, inhibition of the IL-1β to IL-6 to hsCRP pathway achieved by canakinumab (but not methotrexate), appears to play a role critical in ASCVD (19).

A recent trial evaluating low-dose colchicine following MI, COLCOT (Colchicine Cardiovascular Outcomes Trial), demonstrated a reduction in the primary composite outcome of cardiovascular death, cardiac arrest, non-fatal MI, stroke, or angina leading to revascularization (HR 0.77; 95% CI 0.61–0.96; *p* = 0.02) (20). HsCRP was only measured in a subgroup of 207 patients, with a placebo-adjusted mean percent change of −10.1% of hsCRP at 6 months in those randomized to colchicine. The broad use of anti-inflammatories for prevention of cardiovascular events is not yet widely recommended, particularly in those without elevated systemic inflammation, though colchicine may ultimately provide a low-cost intervention for secondary prevention in select patients based on the findings above.

Recently, clonal hematopoiesis of indeterminate potential (CHIP) has emerged as a risk factor for ASCVD through inflammatory pathways (21). CHIP refers to clonal expansion of hematopoietic stem cells due to acquired somatic mutations, which occurs during the aging process. Deficiency of *TET2* (tet methylcytosine dioxygenase 2), one of the genes associated with CHIP, was shown to promote atherogenesis in mouse models through an IL-1β-dependent and NLRP3 inflammasome- dependent mechanism (22). CHIP is surprisingly common, occurring in up to 20% of septuagenarians, and though it rarely transforms to acute leukemia (occurring 0.5–1% per year in carriers), CHIP confers a 40% increased risk of CVD and thus has emerged as a novel cardiovascular risk factor. Intense investigation is underway to determine optimal approaches to recognition and management of this non-traditional ASCVD risk factor (23).

Based on the discussion above as well as multiple observations implicating higher rates of ASCVD in those with chronic inflammatory diseases (e.g., rheumatoid arthritis, systemic lupus erythematosus, psoriasis, human immunodeficiency virus, etc.), there is growing recognition of the importance of the link between inflammation and cardiovascular disease. An important case study of the role of inflammation in ASCVD is the Tsimane, a pre-industrial population in Bolivia. Despite elevated inflammatory markers (51% of participants had a hs-CRP above 3.0 mg/L), this population had a 5-fold lower prevalence of coronary artery calcium than an industrialized population, likely attributed to a low prevalence of traditional risk factors (24). This example emphasizes the importance of traditional risk factors in risk assessment. In the 2019 American College of Cardiology (ACC)/American Heart Association (AHA) Primary Prevention guidelines, chronic inflammatory diseases are considered risk enhancers and may be considered when quantitative risk assessment using traditional risk factors appears to underestimate risk of ASCVD. As of 2019, there are no anti-inflammatory drugs with a formal indication for use in prevention of cardiovascular events, though clearly the inflammatory pathway is an important avenue for further research in cardiovascular disease prevention.

## Residual Thrombotic Risk

### Anticoagulation

The routine use of antiplatelet medications in primary prevention has come under scrutiny given recent data that suggest an unfavorable risk:benefit ratio when used in those without manifest cardiovascular disease (25–28). However, in patients with established ASCVD, antiplatelet agents are an essential component of optimal medical management. Despite appropriate antiplatelet use, usually with aspirin, risk of atherothrombosis and subsequent cardiovascular events remain (29). For example, in the Antithrombotic Trialists' Collaboration, which included 16 secondary prevention randomized trials of 17,000 patients, although aspirin did reduce the risk of serious vascular events, this endpoint still occurred in 6.7% of patients, as compared with 8.2% in the placebo group (30). As a result, there is interest in identification of patients that would benefit from intensification of antithrombotic or anticoagulant therapy.

Two trials have demonstrated the utility of adding low-dose anticoagulation to antiplatelet therapy. In ATLAS ACS 2-TIMI 51 (Anti-Xa Therapy to Lower Cardiovascular Events in Addition to Standard Therapy in Subjects with Acute Coronary Syndrome- Thrombolysis in Myocardial Infarction Trial 51), the addition of low dose rivaroxaban (2.5 or 5 mg twice a day) to antiplatelet therapy after a recent cardiovascular event (with 93% of patients on dual anti-platelet therapy) demonstrated a 16% relative risk reduction in major adverse cardiovascular events (31). However, this benefit was offset by an increase in major bleeding (2.1% vs. 0.6%, *p* < 0.001) and intracranial bleeding.

COMPASS (Cardiovascular Outcomes for People Using Anticoagulation Strategies) demonstrated that low-dose rivaroxaban plus aspirin vs. aspirin alone led to a 24% reduction in the primary outcome and a 22% reduction in the net clinical benefit endpoint (composite of primary outcome, fatal bleeding, and symptomatic bleeding into a critical organ/area) (32). Importantly, in a subgroup analysis, the magnitude of benefit was even more pronounced in those with peripheral arterial disease, a population with increased atherosclerotic burden generally affecting multiple vascular beds, and thus at higher risk of thrombotic events (33). Given these findings low-dose rivaroxaban appears to be a promising avenue for further reduction of residual thrombotic risk.

### Dual Antiplatelet Therapy

Dual antiplatelet therapy (DAPT), with aspirin and a P2Y12 inhibitor, is a mainstay of therapy up to 1 year post percutaneous coronary intervention (PCI). The P2Y12 inhibitors that are used clinically are listed in Table 1. However, the benefit of extending DAPT beyond 12 months has been less clear. PEGASUS-TIMI 54 (Prevention of Cardiovascular Events in Patients with Prior Heart Attack Using Ticagrelor Compared to Placebo on a Background of Aspirin–Thrombolysis In Myocardial Infarction 54) demonstrated that the addition of ticagrelor to aspirin resulted in a 16% reduction in MACE (34). A meta-analysis of 6 trials evaluating the efficacy of long-term DAPT in the post-ACS stable cardiovascular disease setting demonstrated similar results (35). However, the benefits of prolonged DAPT were offset by an increased risk of major (but not fatal) bleeding.

**Table 1:** P2Y12 inhibitors in clinical use.

	**Dose**	**Pharmacokinetics**	**Contraindications**	**Adverse effects**
Clopidogrel	300 or 600 mg loading dose followed by 75 mg daily for maintenance	Prodrug that is metabolized to a pharmacologically active metabolite	Anaphylactic reaction	Generally well-tolerated but may have an allergic reaction with diffuse urticaria
Prasugrel	60 mg loading dose followed by 10 mg daily for maintenance	Prodrug that is metabolized to a pharmacologically active metabolite and inactive metabolites	**Absolute contraindication:** history of stroke or TIA **Relative contraindication:** ≥75 years of age or patient weight <60 kg	Generally well-tolerated. Like clopidogrel may have diffuse urticaria
Ticagrelor	180 mg loading dose followed by 80 mg twice daily for maintenance	Not a prodrug and does not require bioactivation	Anaphylactic reaction	Dyspnea in 14%

THEMIS (The Effect of Ticagrelor on Health Outcomes in Diabetes Mellitus Patients Intervention Study) and its substudy in prior percutaneous coronary intervention (PCI) or coronary bypass patients, THEMIS-PCI, was presented at the European Society of Cardiology Congress in 2019. This study sought to identify populations that would receive greater benefit from DAPT (36). In patients with stable ischemic heart disease and diabetes, the addition of ticagrelor to aspirin in diabetic patients led to a reduction in MI (2.8% with ticagrelor plus aspirin vs. 3.4% with aspirin plus placebo). An exploratory composite outcome of irreversible harm, all-cause death, MI, stroke, fatal bleeding, or intracranial hemorrhage occurred in 10.1% of the ticagrelor + aspirin group compared with 10.8% of aspirin + placebo group. There was no difference in cardiovascular death, but the incidence of TIMI major bleeding was higher in the ticagrelor + aspirin group as compared to the aspirin + placebo group (2.2% vs. 1.0%, respectively, *p* < 0.001). THEMIS-PCI enrolled patients with diabetes and stable CAD with a history of PCI or coronary artery bypass, and thus enriched for a population that had tolerated DAPT previously (37). In this select population, the investigators demonstrated that fewer patients in the treatment arm suffered from the composite endpoint of cardiovascular death, MI, or stroke [7.3% in ticagrelor + aspirin arm vs. 8.6% in aspirin + placebo arm; HR 0.85 (95% CI 0.74–0.97), *p* = 0.013]. There was statistically significant higher occurrence of TIMI major bleeding in the treatment arm [HR 2.03 (95% CI 1.48–2.76), *p* < 0.0001] but fatal bleeding and intracranial hemorrhage did not differ between the two groups. Interestingly, ticagrelor improved net clinical benefit in patients with prior history of PCI (e.g., those more likely to have received DAPT previously).

## Residual Metabolic Risk

### Lipoprotein (a)

Lipoprotein (a) (Lp[a]) is an LDL-like particle covalently bound to apolipoprotein(a) [apo(a)], a non-functional mimic of plasminogen (38, 39). Lp(a) plays an active role in vascular atherothrombosis and its plasma levels are highly heritable (40). In the INTERHEART study, an Lp(a) level >50 mg/dL was associated with an increased risk of MI (OR 1.48, 95% CI 1.32–1.67, *p* < 0.001) (41). A subanalysis from JUPITER demonstrated that Lp(a) was a strong predictor of residual risk in patients already on a statin (adjusted HR 1.27; 95% CI 1.01–1.59, *p* = 0.04), independent of LDL-C and other risk factors (42). Whether Lp(a) should be a target of therapy remains a matter of debate. While the epidemiology and genetics suggest that Lp(a) is causal for ASCVD and calcific aortic stenosis (43–46), no randomized controlled trials have been performed to answer this question. Mendelian randomization studies demonstrate lower risk of ASCVD proportional to reduction in genetically conferred Lp(a) levels (46, 47), and genome-wide association studies demonstrate that single-nucleotide polymorphisms associated with the *LPA* locus are associated with ASCVD (48, 49). In FOURIER, evolocumab reduced Lp(a) levels by a median of 26.9%, which was moderately correlated with change in LDL-C (*r* = 0.37) (50). In patients with above the median baseline Lp(a), evolocumab was associated with a 23% reduction in the risk of CHD death, MI or urgent revascularization (compared to placebo), whereas only a 7% reduction was seen in those with Lp(a) below the median (P for interaction = 0.07). Importantly, in a *post-hoc* analysis of phase 3 ODYSSEY trials, Lp(a) reductions were not significantly associated with MACE reductions independently of LDL-C, except at the highest baseline values of Lp(a) (≥105 nmol/L), in whom reductions of Lp(a) using alirocumab translated to significant reductions in risk beyond LDL-C lowering (51, 52). Based on the available data, the AHA/ACC guideline on blood cholesterol recommend initiating moderate- to high-intensity statin therapy in adults aged 40–75 years with a 10 year ASCVD risk of 7.5% to ≤ 20% and Lp(a) ≥100 nmol/L. Additionally, high risk patients with LDL-C ≥70 mg/dL (non-HDL-C ≥100 mg/dL) and a Lp(a) ≥100 nmol/L on maximally tolerated statin should be considered for more intensive therapies (ezetimibe and PCSK9 inhibitors) to lower LDL-C (53).

Currently, novel therapies that selectively target Lp(a) are under development. A phase 2 trial of AKCEA apo(a)-LRx, an apo(a) antisense oligonucleotide, reduced Lp(a) up to 80% (54). A phase 3 study is being planned. Additionally, a therapeutic monoclonal antibody targeting oxidized phospholipids [of which Lp(a) represents the major plasma carrier] that binds and inactivates the pro-osteogenic activity of Lp(a) demonstrated promising *in vitro* data (55, 56). The cardiovascular outcomes trials of these novel therapies are awaited with interest.

### Triglycerides and Remnant Cholesterol

Triglycerides and triglyceride-rich lipoproteins (TGRL) have also been identified as important contributors to residual risk. TGRLs are derived from the diet (chylomicrons and their remnants) and the liver (VLDL and their remnants) and circulate in the plasma (57). Lipoprotein lipase (LPL) lines the luminal surface of capillaries and hydrolyzes the TGs within the core of these TGRLs to free fatty acids (FFA) and glycerol. As FFA are liberated, the TGRL particles are remodeled physically (become smaller by losing TG and surface phospholipids) and chemically (become relatively cholesterol enriched). These partially lipolyzed TGRL are known as remnant particles (58).

Several studies have demonstrated an epidemiologic association between TGRLs and ASCVD. Additionally, a causal role was supported by Mendelian randomization studies (59). Moreover, remnant cholesterol, calculated as total cholesterol minus HDL-C minus LDL-C, consistently demonstrates an association with cardiovascular disease and mortality (60–62). TGRLs and their remnants are thought to be atherogenic through their ability to readily penetrate the arterial wall and direct uptake by arterial wall macrophages without further modification, in contrast to the oxidative modification required by arterial macrophages to take up LDL (63). Additionally, increased oxidative stress, impairment of endothelium-dependent vasodilation, activation of inflammation, and activation of the pro-thrombotic factors are all possible mechanisms explaining the pathogenicity of TGRLs (64–68).

There has been debate about the role of targeting triglycerides to improve cardiovascular outcomes. Trials with several triglyceride lowering therapeutics demonstrated inconsistent results (69–75). However, the use of high-dose, purified eicosapentaenoic acid (EPA) in populations with elevated triglycerides suggest a role in reducing residual cardiovascular risk. In JELIS (Japan EPA Lipid Intervention Study), an open-label blinded study of supplementation with 1.8 g/day of EPA and a statin (pravastatin 10 mg or simvastatin 5 mg) vs. statin alone in a primary prevention population, the investigators demonstrated a statistically significant (*p* = 0.01) 19% reduction in major coronary events (76). In a sub-analysis of JELIS evaluating individuals with triglycerides >150 mg/dL and a HDL-C <40 mg/dL, EPA treatment led to a large reduction in incident coronary artery disease (HR 0.47; 95% CI 0.23–0.98; *p* = 0.043), highlighting the potential benefit of this therapy from a primary prevention standpoint in individuals with elevated triglycerides and low HDL-C (76). Recently, the REDUCE-IT (Reduction of Cardiovascular Events With EPA–Intervention) trial enrolled patients with established ASCVD or diabetes with other risk factors and mild-moderate hypertriglyceridemia (77). All patients were on background statin therapy and had fasting triglyceride levels of 135 to 499 mg/dL and LDL-C levels of 41 to 100 mg/dL. Subjects were randomized to 4 grams of EPA daily or a mineral oil placebo. The intervention arm exhibited a 25% relative risk reduction in the primary composite endpoint of cardiovascular death, non-fatal MI, non-fatal stroke, coronary revascularization, or unstable angina (17.2% in the EPA group vs. 22.0% in the placebo group; HR 0.75, 95% CI 0.68–0.83; *p* < 0.001). Interestingly, the large reduction in the primary end-point was accompanied with only a modest decrease in plasma triglyceride concentration (median 18.3%, 39 mg/dL), suggesting that there may be additional mechanisms (e.g., antithrombotic, antiarrhythmic, antioxidant, anti-inflammatory, etc.) beyond triglyceride-lowering that lead to the improvement in cardiovascular outcomes. Moreover, the reduction in ASCVD events was similar regardless of whether or not triglycerides were reduced to below 150 mg/dL, providing further evidence supporting the effects were due to factors other than reduction in triglycerides. Importantly, STRENGTH (Outcomes Study to Assess Statin Residual Risk Reduction with EpaNova in HiGh CV Risk), a trial investigating the use of a Epanova, a formulation consisting of EPA + docoseahexaenoic acid, was stopped due to futility, suggesting that EPA itself may be the important component for risk reduction, as shown in REDUCE-IT.

### High-Density Lipoprotein-Cholesterol

One of the most strikingly consistent relationships in cardiovascular epidemiology relates to the association of low levels high-density lipoprotein (HDL)-cholesterol (HDL-C) and ASCVD (78–80). In the Framingham cohort, there was a graded decrease in risk for every 1 mg/dL increase in HDL-C concentration (81). Although there had been hope that raising HDL-C levels would reduce cardiovascular risk, randomized controlled trials with niacin and cholesterol ester transfer protein inhibitors, while effective at raising HDL-C, did not lead to a reduction in major adverse cardiovascular events (82–87). Additionally, Mendelian randomization studies have not supported a causal relationship between HDL-C levels and cardiovascular disease (88, 89). To confuse the matter further, very high levels of HDL-C associate with poorer outcomes in large general population studies in Canada and Denmark (90, 91). The evolution in thinking suggests that functional measures of HDL may be more clinically useful than concentration. As proof of principle, one functional measurement of HDL, cholesterol efflux capacity, demonstrated the ability to predict both prevalent and incident coronary artery disease (92–95). In the Dallas Heart Study, in 2,924 patients without known ASCVD, there was a 67% reduction in cardiovascular risk in those in the highest quartile of cholesterol efflux capacity vs. the lowest quartile (hazard ratio, 0.33; 95% CI, 0.19–0.55). Importantly, cholesterol efflux capacity exhibited a weak correlation with HDL-C level (Spearman correlation coefficient of 0.07, *p* < 0.05). At present, low HDL-C (and perhaps at very high levels) does serve to identify patients at risk for adverse events, though at present no pharmacological treatment targeting HDL-C has proven effective in cardiovascular reducing risk. Currently, there are ongoing investigations with apolipoprotein A-1 infusion therapies to test if raising and/or improving the function of HDL can reduce residual risk, where prior efforts in raising HDL-C concentration had failed (96).

### Diabetes

For years, none of the medications used to treat diabetes demonstrated the ability to reduce cardiovascular events and/or mortality. The advent of the glucagon like peptide 1 receptor agonists (GLP-1RA) and sodium-glucose cotransporter 2 inhibitors (SGLT2i) heralded a new era in diabetes care. EMPA-REG OUTCOME (Empagliflozin, Cardiovascular Outcomes, and Mortality in Type 2 Diabetes trial) in 2015 ushered in a paradigm shift in the treatment of diabetes and cardiovascular disease (97). Since that time, there have been a number of other trials that demonstrated improvement in cardiovascular outcomes with these two classes of drugs (Table 2) (98–102). An important consideration is that the substantial benefits seen with these medications occurred in a cohort in which a majority of patients were on optimal or near optimal background therapy per current guidelines. As an example, in EMPA-REG OUTCOME, 81% of subjects were on angiotensin-converting enzyme inhibitors (ACE-i) or angiotensin receptor blockers (ARB), 77% were on statins, and 82% were on aspirin. Despite this, in the placebo arm, the primary composite outcome of cardiovascular death, non-fatal MI, or non-fatal stroke occurred in 12.1% of the group, with 5.9% dying from cardiovascular causes over the 3 year observation time. Although empagliflozin was heralded as a great success for a 14% relative risk reduction of the primary outcome and a striking 38% relative risk reduction for cardiovascular death, these reductions were still associated with a significant proportion (10.5%) of participants sustaining major adverse cardiovascular events, with nearly 4% succumbing to death from cardiovascular causes. Although these trials signal an important advancement in the care of patients with diabetes, they also serve to highlight the substantial residual risk that exists despite adherence to standard of care therapies.

**Table 2:** Cardiovascular outcomes in select randomized controlled trials of new diabetes therapies.

**Trial**	**Drug**	**Drug Class**	**Number of patients**	**Median duration (years)**	**Hgb A1C reduction (%)**	**Primary endpoint**	**CV death**	**MI**	**HF hospitalization**	**All-cause mortality**,
EMPA-REG	Empagliflozin	SGLT2-i	7,020	3.1	0.24–0.36 (10 and 25 mg empagliflozin, respectively)	3-point MACE HR 0.86 (95% CI 0.74–0.99)	HR 0.62 (0.49–0.77)	HR 0.87 (0.70–0.95)	HR 0.65 (0.50–0.85)	HR 0.68 (0.57–0.82)
CANVAS	Canagliflozin	SGLT2-i	10,142	2.4	0.58	3-point MACE HR 0.86 (95% CI 0.75–0.97)	HR 0.87 (0.72–1.06)	HR 0.89 (0.73–1.09)	HR 0.67 (0.52–0.87)	HR 0.87 (0.74–1.01)
DECLARE-TIMI 58	Dapagliflozin	SGLT2-i	17,160	4.0	0.42	Composite of CV death or HHF HR 0.83 (95% 0.73–0.95) MACE HR 0.93 (95% CI 0.84–1.03; *p =* 0.17)	*Without reduced EF:* HR 1.08 (0.89–1.31) *With reduced EF:* HR 0.55 (0.34–0.90)	HR 0.89 (0.77–1.01)	*Without reduced EF:* HR 0.76 (0.62–0.92) *With reduced EF:* HR 0.64 (0.43–0.95)	*Without reduced EF:* HR 0.97 (0.86–1.10) *With reduced EF:* HR 0.59 (0.40–0.88)
CREDENCE	Canagliflozin	SGLT2-i	4,401	2.6	0.25	Composite of end-stage kidney disease, doubling of serum creatinine, death from renal or CV causes HR 0.66 (95% CI 0.53–0.81)	HR 0.78 (0.61–1.00)	HR 0.80 (0.67–0.95) (composite of CV death, MI, or stroke)	HR 0.61 (0.47–0.80)	HR 0.83 (0.68–1.02)
DAPA-HF	Dapagliflozin	SGLT2-i	4744	1.5	0.21	Composite of worsening heart failure or death from CV causes HR 0.74 (95% CI 0.65–0.85)	HR 0.82 (95% CI 0.69–0.98)	–	HR 0.70 (95% CI 0.59–0.83)	HR 0.83 (95% CI 0.71–0.97)
LEADER	Liraglutide	GLP-1ra	9,340	3.8	0.40	3-point MACE HR 0.87 (95% CI 0.78–0.97)	HR 0.78 (0.66–0.93)	HR 0.86 (0.73–1.00)	HR 0.87 (0.73–1.05)	HR 0.85 (0.74–0.97)
SUSTAIN-6	Semaglutide	GLP-1ra	3,297	2.1	0.7–1.0 (0.5 mg and 1.0 mg semaglutide, respectively)	3-point MACE HR 0.74 (95% CI 0.58–0.95)	HR 0.98 (0.65–1.48)	HR 0.74 (0.51–1.08)	HR 1.11 (0.77–1.61)	HR 1.05 (0.74–1.50)
HARMONY	Albiglutide	GLP-1ra	9,463	1.5	0.52	3-point MACE HR 0.78 (95% CI 0.68–0.90)	HR 0.93 (0.73–1.19)	HR 0.75 (0.61–0.90)	HR 0.85 (0.70–1.04) (composite of CV death and HF hospitalization)	HR 0.95 (0.79–1.16)

*Hgb A1C, hemoglobin A1C; MACE, major adverse cardiovascular event; HHF, hospitalization for heart failure; 3-point MACE: CV death, non-fatal MI, non-fatal stroke; CV, cardiovascular; MI, myocardial infarction; HF, heart failure, HR, hazard ratio; GLP-1ra, glucagon like peptide 1 receptor agonist; SGLT2-i, sodium-glucose cotransporter 2 inhibitor; EF, left ventricular ejection fraction*.

Likely, there are two different mechanisms of benefit between the two classes. SLGT2i exhibit their benefit early on following initiation and the benefit is seen with heart failure hospitalizations and significant slowing of decline in renal function. The possible explanations for these observations include osmotic diuresis leading to improved cardiac hemodynamics by reduction in left ventricular preload, lowering of body weight due to calorie and fluid losses, and lowering of blood pressure (103, 104). Another proposed mechanism of cardiovascular benefit may be a shift in fuel energetics from free fatty acids to ketones, which are a preferred substrate for myocardial cells (105). This improvement in metabolic efficiency is theorized to translate to cardiovascular benefit. GLP1-RA display their mortality benefit following a matter of months to years on atherosclerotic outcomes and do not appear to have a significant impact on heart failure endpoints. The mechanism for the anti-atherogenic effect of GLP1-RA is unclear, but the reduction of blood pressure, weight loss, and avoidance of hypoglycemia associated with these medications may contribute to improved cardiovascular outcomes (106).

Importantly, SGLT2i demonstrate benefit in individuals without diabetes as well, particularly in those with heart failure and reduced ejection fraction as demonstrated in the DAPA-HF study (Study to Evaluate the Effect of Dapagliflozin on the Incidence of Worsening Heart Failure or Cardiovascular Death in Patients with Chronic Heart Failure) (107). The application of the SGLT2i in patients without diabetes but with heart failure is an exciting area of active investigation.

As stated previously, an important consideration is that these novel medications displayed additive benefit to background therapy of proven cardioprotective medications (i.e., statins, ACEi/ARB). In a meta-analysis of over 18,000 patients with diabetes treated with statins, for each mmol/L (39 mg/dL) reduction in LDL-C due to statin therapy, there was a proportional 9% reduction in mortality and a 13% reduction in vascular mortality, irrespective of prior history of cardiovascular disease (108). As such, at least a moderate-intensity statin is indicated in all individuals with diabetes aged 40–75 years of age, with a high-intensity statin indicated for those with ASCVD or at high risk for ASCVD (10 year ASCVD risk ≥ 20%) (53, 109). Similarly, ACE-i/ARBs have demonstrated consistent reduction in cardiovascular events in hypertensive patients with diabetes, particularly in those with diabetic nephropathy, and as such are indicated in this population (13, 110). There is also an updated recommendation from the American Diabetes Association for use of EPA in patients with diabetes and cardiovascular disease or with risk factors on a statin with controlled LDL-C but elevated triglycerides (135–499 mg/dL) based on the strength of evidence presented above in REDUCE-IT (77, 111). As new therapies emerge that serve to incrementally reduce risk in this high-risk population, it is critical to ensure adherence to proven preventative therapies.

## Conclusion

Identification and treatment of residual cardiovascular risk is critical to optimize patient outcomes, particularly in those at risk for recurrent events despite optimal treatment of traditional risk factors. The pathways discussed in this review represent sources of residual risk for the clinician to be mindful of when personalizing risk prediction and subsequent treatment for each patient. As we reach the limits of benefit of currently available therapies, it will be important to investigate and await the results of new approaches to managing residual cardiovascular risk. In the meantime, it seems prudent to recognize emerging risk factors and adopt new therapeutics that address some of these risk factors (i.e., EPA, SGLT2i/GLP1-ra, low-dose rivaroxaban).

## Author Contributions

All authors listed have made a substantial, direct and intellectual contribution to the work, and approved it for publication.

## Conflict of Interest

The authors declare that the research was conducted in the absence of any commercial or financial relationships that could be construed as a potential conflict of interest.
